# New Paradigm of Machine Learning (ML) in Personalized Oncology: Data Trimming for Squeezing More Biomarkers From Clinical Datasets

**DOI:** 10.3389/fonc.2019.00658

**Published:** 2019-07-17

**Authors:** Nicolas Borisov, Anton Buzdin

**Affiliations:** ^1^Department of Personalized Medicine, I.M. Sechenov First Moscow State Medical University (Sechenov University), Moscow, Russia; ^2^Department of Genomics and Postgenomic Technologies, Shemyakin-Ovchinnikov Institute of Bioorganic Chemistry, Moscow, Russia; ^3^Department of Bioinformatics and Molecular Networks, OmicsWay Corporation, Walnut, CA, United States

**Keywords:** bioinformatics, personalized medicine, oncology, P vs. N problem, machine learning

## Introduction

Personalized medicine has a huge potential of transforming healthcare standards when selection of therapies according to standard guidelines often fails, which can be the case in oncology (1, 2), endocrinology (3, 4), neurology (3), treatment of infectious diseases (5, 6) and hemostatic disorders (7, 8). Nowadays, personalized approach can be based on a solid fundament of big biomedical data obtained for an individual patient, analyzed vs. comparable datasets for other individual cases with known clinical outcome. This can help, for example, developing new criteria for predicting response of a cancer patient to a certain treatment.

The analysis of Big Data in oncology can benefit significantly from being empowered by *machine learning* (ML) techniques (9–13) tailored for solving this “*P* vs. *N”* problem. ML is usually defined as the study of algorithmically-built mathematical models that have been fitted for the portion of data called the *training dataset*, to make predictions for the similarly-obtained and similarly structured data called the *test* or *validation dataset*. Major principles of ML have been formulated more than half a century ago and transformed methodology in many areas such as engineering, physics, banking, defense, agriculture, and meteorology (11, 14). Efficiencies of ML-based predictor/classifier models are described by specific quality metrics such as sensitivity (Sn), specificity (Sp), area under ROC curve (AUC), accuracy rate (ACC), Matthews correlation coefficient (MCC), or by *p*-values from statistical tests distinguishing one class from another (15).

**Figure 1:**
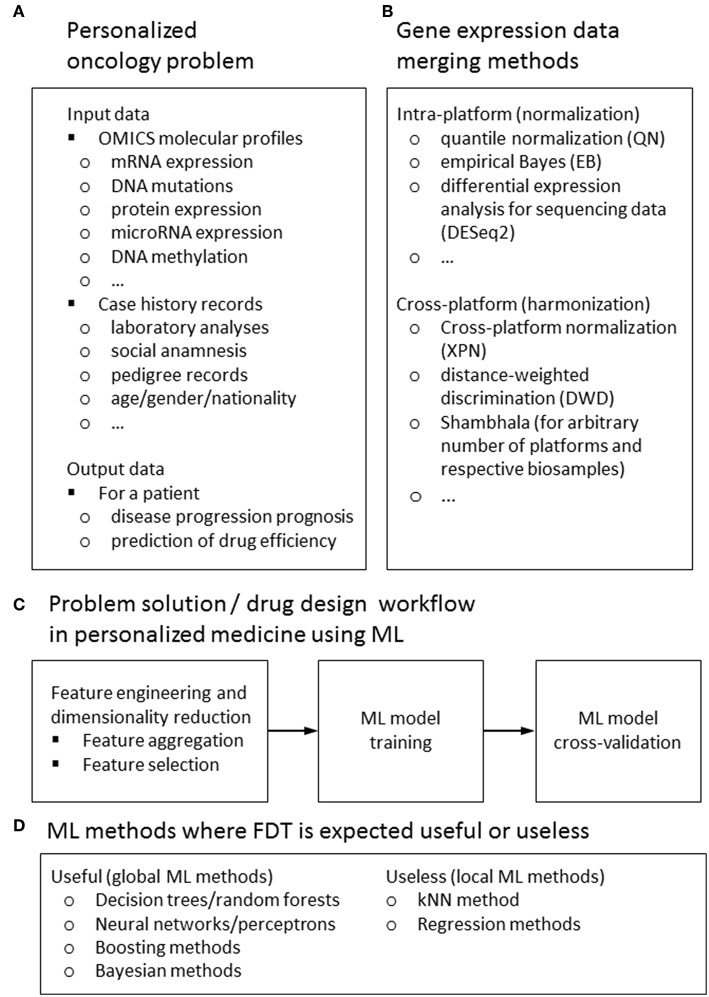
Input and output data types **(A)** methods for feature harmonization **(B)** general workflow **(C)** for a ML-assisted solution of typical problem in personalized medicine; ML methods for those FDT is expected to be useful or useless **(D)**.

However, it was only in the beginning of XXI century when such ML on Big Data became possible in biomedicine, still not having a groundbreaking effect (16). This delay is most probably due to relatively recent emergence of experimental methods generating big biomedical data connected with the sufficiently developed IT infrastructure. Among those game-changing experimental methods the major role was played by next-generation sequencing (NGS) and novel mass-spectrometry approaches which enabled performing whole genome-, transcriptome-, proteome-, and metabolome analyses relatively fast and cheap (17–19), see Figure 1A. This allowed to feed ML methods with big biomedical data thus generating beneficial outputs, also in the field of clinical medicine. For example, over 150 scientific papers have been indexed in the PubMed repository during last 24 months mentioning *machine learning* and *drug sensitivity*^1^.

Here we will focus on applying ML for personalized medicine, primarily oncology, dealing with attempts to generate as much as possible treatment response biomarkers from mediocre datasets. From the point of view of classical ML approaches, most if not all of the available clinical genetic datasets are insufficient for solving the task of differentiating, e.g., treatment responders from non-responders (9, 20). Numbers of features measured by NGS (e.g., mutations or gene expression values) are far greater than numbers of individual patients with traced clinical outcomes involved in each respective dataset. To generate statistically significant predictions, this requires extensive reduction of a pool of features to be considered, to make their number not exceeding the number of individuals analyzed (16). To increase the number of individuals, the datasets can be merged using cross-dataset harmonization. Different methods can be used to harmonize data obtained using the same (21, 22) or different experimental platforms (23, 24), or even using multiple platforms (25) (Figure 1).

## ML Input Data and Workflow

For ML applications dealing with prediction of patient's individual response(s) on drugs and different treatment regimens, two types of data are most frequently used (Figure 1A):

1. Various multi-omics data, i.e., mRNA, microRNA, and protein expression levels, mutations in genomic DNA and epigenetic profiles (primarily DNA methylation) (26, 27). These data may be compared with the analogous types of data obtained on cell cultures in relation to sensitivity to therapeutics/treatment regimens, such as the data taken from the Broad Institute (28) and CancerRxGene (29) projects. These examples include, respectively, either changes of gene expression profiles influenced by the addition of drugs to cell culturing media, or gene expression and polymorphism/mutation profiles for many cell lines linked with their measured sensitivities to cancer drugs (30). These datasets are regarded plausible models for training ML drug sensitivity classifiers because they have thousands of individual “cases”—pairs cell culture/drug, each profiled in several replicates.
2. Alternatively, other types of data can be used including gender, age, results of clinical laboratory tests, functional diagnostics data (ECG, EEG etc.), risk factors, social anamnesis, and other electronic health records.

A typical workflow of ML drug sensitivity assay includes the following steps (Figure 1C):

1. *Data reduction, feature selection, and building on the training dataset*. Usually, in the collected raw data, the number of features (*NF*) exceeds the number of cases (*NC*), so that to provide a robust ML model, one must reduce the data to make the number of selected features (*NS*) lower than *NC* or at least comparable to it. This goal can be achieved in several ways. The raw data may be aggregated, e.g., in molecular pathways (2); or co-expressed/co-mutated clusters (31). Sometimes, the co-expression- and pathway topology-based analysis may be combined (32).Alternatively, they can be filtered according to specific functional of statistical traits (e.g., only the genes coding for tyrosine kinases are left; or genes with the highest abilities to discriminate responders from non-responders in training datasets) (33). The statistical methods for feature selection may include Pearson chi-squared test (34) or correlation test (34, 35). Other options are variance thresholding (VT), genetic algorithms (36), univariate feature selection (UFE), recursive feature elimination (RFE), principal component analysis (PCA) (35), CUR matrix (37) decomposition (27) and covariate regression (38).
2. *Applying ML algorithm*. The following methods may be used: support vector machines, SVM (2, 27, 39), *k* nearest neighbors, kNN (39), decision trees, DT (34, 39) or random forest, RF (39, 40). Alternatively, one can use artificial neural network, ANV (39), elastic net (41), back propagation networks (42), naïve Bayesian (27), logistic (27, 39), penalized (43), and lasso (43) regression models. In some cases, the hybrid global-local approaches, like combination of decision trees, random forests/SVM with kNN are used (2, 33, 39, 44, 45).
3. *Cross-validation and performance quality check*. The data obtained with the training dataset are then validated using independent validation dataset. For the cross-validation of machine learning methods, 5- or 10-fold cross validations are most commonly used. For datasets with smaller number of preceding cases (*NC*) the leave-one-out (LOO) scheme is preferable (2, 33, 43).

## Shifting the Paradigm

The demonstrated performance of ML classifiers was high for problems like age recognition based on biochemical markers (41), but significantly lower for predictions of drug response in cancer patients (27, 46), with the exception of few reports based on very small patient cohorts (43).

A new paradigm recently emerged of considering flexible rather than fixed sets of features that are fitted individually for every comparison of a biosample with the pool of controls/training datasets (33). This can be done by means of *data trimming*^2^—sample-specific removal of features. The irrelevant features in a sample that don't have significant number of neighboring hits in the training dataset are removed from further analyses. In a pilot application for the SVM method of ML and high throughput gene expression data, this enabled to dramatically increase number and quality of biomarkers predicting responses to chemotherapy treatments for 10/10 cohorts of 46–235 cancer patients (33). Among them, in 3/10 cases basic ML applications were impossible to generate biomarkers of a sufficient quality.

The application of *flexible data trimming* (FDT) procedure prevents ML classifier from extrapolation by excluding non-informative features. Contrary to other complex data transfer techniques, this approach is heuristic, based on a common geometrical sense. For each point of a validation dataset, it takes into account only the proximal points of the training dataset. Thus, for every point of a validation dataset, the training dataset is adjusted to form a floating window. That was why we called (33) our FDT method *FLOating Window Projective Separator* (*FloWPS*).

## Discussion

Certainly, FloWPS is not the only possible method of data reduction for ML in oncology. In the pilot study, a simple PCA-based alternative was tried, which was less successful (33).

One of the major limitations of FloWPS is that it can be time-consuming at the level of optimization of data trimming parameters. The required computational time for such optimization grows cubically with the number of preceding cases in the training dataset. For example, for a 31 Gb RAM and 8·4.20 GHz CPUs computer running the Python FloWPS code for a dataset of 46 samples (33) takes ~20 s, whereas for a bigger dataset of 235 samples (33) it requires already few hours.

SVM is one of the most popular methods of ML nowadays (9, 48). However, using data trimming procedure has dramatically improved its performance for the task of classification cancer drug responders and non-responders. This means that it can be highly beneficial for the other ML methods as well. The FDT method simultaneously combines the advantages of both global (like SVM) and local (like kNN) methods of ML, and successfully acts even when purely local and global approaches fail. Due to its hybrid (global + local) nature, we expect that FloWPS may be also effective for other global ML methods such as decision trees/random forests, neural networks/multi-layer perceptrons, decision trees/random forests and boosting or Bayesian methods for ML, but may be useless for purely local approaches such as kNN or regression models (Figure 1D).

In its first published application, the data trimming could operate with high throughput gene expression or mutation profiles (33). However, it can be used for any type of Big Data in biomedicine, but not only. In this opinion paper, we speculate that this new concept has a potential to broadly introduce the use of ML in personalized oncology and, possibly, significantly expand its presence in many other fields.

## Availability of Code

The R package flowpspkg.tar.gz for FloWPS method and README manual are available at GitLab through the link: https://gitlab.com/borisov_oncobox/flowpspkg.

## Author Contributions

All authors listed have made a substantial, direct and intellectual contribution to the work, and approved it for publication.

### Conflict of Interest Statement

The authors declare that the research was conducted in the absence of any commercial or financial relationships that could be construed as a potential conflict of interest.

## Footnotes

^1^This is the result of a PubMed query: https://www.ncbi.nlm.nih.gov/pubmed/?term=machine+learning+drug+sensitivity

^2^Data trimming is the process of removing or excluding extreme values, or outliers, from a dataset (47).
